# Supplementary material for: Methodological challenges using routine clinical care data for real-world evidence: a rapid review utilizing a systematic literature search and focus group discussion
Source: BMC Med Res Methodol. 2025 Jan 14;25:8. doi: 10.1186/s12874-024-02440-x (PMC11731536; doi:10.1186/s12874-024-02440-x)
Supplement: Supplementary file 1 — Supplementary Material 1. [file 12874_2024_2440_MOESM1_ESM.docx]

## Supplementary S1

Table S1 Search strategy

| 1. In Title and Abstract |  | 1. Journals | 1. Time |
| --- | --- | --- | --- |
| real-world evidence **OR** |  | The New England journal of medicine **OR** |  |
| Real-world data **OR** |  | The Lancet. Oncology **OR** |  |
| real-world **OR** |  | BMJ (Clinical research ed.) **OR** |  |
| RWE **OR** |  | JAMA **OR** | Filter: |
| routine data **OR** | **AND** | Journal of clinical oncology: official journal of the American Society of Clinical Oncology **OR** | 2018 – 2023 |
| routine care data **OR** |  | JAMA oncology **OR** |  |
| Emulation **OR** |  | Lancet (London, England) |  |
| Electronic health record |  |  |  |

Table S2 Detailed Search History PubMed on October 31st 2023

| Search | Query | Results |
| --- | --- | --- |
| #1 | **Search:** ("The New England journal of medicine"[Journal]) OR ("BMJ (Clinical research ed.)" [Journal]) OR ("The Lancet. Oncology" [Journal]) OR ("JAMA" [Journal]) OR ("Journal of clinical oncology : official journal of the American Society of Clinical Oncology" [Journal]) OR ("JAMA oncology"(Journal]) OR ("Lancet (London, England)"[Journal]) | 435,802 |
| #2 | **Search:** "real-world evidence" [tiab] OR "Real-world data" [tiab] OR real-world[tiab] OR RWE[tiab] OR "routine data" [tiab] or "routine care data " [tiab] OR Emulation [tiab] OR "Electronic health record" [tiab] | 103,854 |
| #3 | **Search:** #1 AND #2 | 387 |
| #4 | **Search:** #1 AND #2 **Filters:** from 2018-2023 | 227 |
