# Supplementary material for: Methodological challenges using routine clinical care data for real-world evidence: a rapid review utilizing a systematic literature search and focus group discussion
Source: BMC Med Res Methodol. 2025 Jan 14;25:8. doi: 10.1186/s12874-024-02440-x (PMC11731536; doi:10.1186/s12874-024-02440-x)
Supplement: Supplementary file 2 — Supplementary Material 2. [file 12874_2024_2440_MOESM2_ESM.docx]

## Supplementary S2

Table S3 Glossary of terms including RWD-specific indication

| Category | Definition | RWD |
| --- | --- | --- |
|  |  |  |
| Confounding | Confounding appears if the confounding variable has a (potentially) causal relationship with both intervention/exposition and the outcome. Includes confounding by indication and residual confounding (unmeasured confounding). [19] |  |
| Selection Bias | Bias introduced by the selection of the reference group. Including racial, survivor and volunteer bias. [19] |  |
| Information Bias | Misclassification of the intervention status or outcomes, or measurement error in outcomes, can lead to information bias. [19] |  |
| Reporting Bias | Bias due to the selection of the reported results [19] |  |
| Publication Bias | A certain type of reporting bias, where the publication of research findings depends on the results. [19] |  |
|  |  |  |
| Coding Challenges | Heterogeneity in coding practices, also with respect to upcoding, or coding systems | x |
| Operationalization or Availability of Variables | Variables are not available in the EHR-system to the extend or in the form needed for research. This may lead to potential unmeasured confounding or selection bias. | x |
| Missing Data | Missing records within variables available in the EHR-system. E.g. not all laboratory variables will be measured at each visit because they might be irrelevant for routine care. | x |
| Follow-up Challenges | Handling different lengths of follow-ups, limited follow up due changes over time |  |
| Validation & Data Quality | Challenges in validation of EHR-Data and changing data quality over time, e.g. due to ongoing digitalization. | x |
|  |  |  |
| Burden of Disease challenges* | Missing information due to death |  |
|  | Heterogeneity and specialization of hospitals |  |
|  | Calibration of algorithms |  |
| Safety and Risk Group challenges* | Case vs. patients: a patient has multiple cases associated with them in routine clinical data. Specifying whether the study is on case- or patient-level. | x |
|  | Reverse p-hacking: intentionally favoring non-significant results |  |
|  | Missing information regarding the reason for therapy switch | x |
|  | Treatment pathways heterogeneity |  |
|  | Off-label medication use | x |
| Treatment Comparison challenges* | Sector boundaries and data availability: challenges in data transferability between different data sectors | x |
|  | Uncertainty about timing of intervention | x |
|  | Control group definition | x |
|  | Verifiability of inclusion and exclusion criteria | x |
|  | Follow up duration- specifically with regards to the lag of efficacy in drugs |  |
| Added General Limitations | Record Linkage: the linkage of different data sources (e.g. registries, biobanks, etc.) or other hospitals/medical facilities | x |
|  | Selection of measurements | x |
|  | Case overlap and data overlap when using different data bases | x |
|  | Estimator vs. Estimand: differentiation and meaning of these terms. |  |

*Challenges that were added by the focus group discussion, Abbreviations: EHR…Electronic Health Records
