# Supplementary material for: Methodological challenges using routine clinical care data for real-world evidence: a rapid review utilizing a systematic literature search and focus group discussion
Source: BMC Med Res Methodol. 2025 Jan 14;25:8. doi: 10.1186/s12874-024-02440-x (PMC11731536; doi:10.1186/s12874-024-02440-x)
Supplement: Supplementary file 3 — Supplementary Material 3. [file 12874_2024_2440_MOESM3_ESM.docx]

## Supplementary S3

Table S4 provides a comprehensive overview of the extracted studies, including their characteristics and limitations described in their respective discussion section. Note, that for some publications (indicated with an asterisk) the category could not be clearly assigned, especially in the case of *Treatment Comparison* and *Safety and Risk Group Analysis*. These publications did not only compare the effectiveness of treatments but specifically compared the risk of adverse events between two or more treatments [37, 40, 43-45, 48, 51].

All studies categorized into the Burden of Disease scenario were cohort studies except for three studies [24, 27, 30] that were developing or validating a machine learning model to predict certain endpoints using EHR data. Three studies [28, 31, 32] examined time-to-event outcomes using Cox regression as well as Kaplan-Meier estimator partly with inverse propensity weights. Prevalence and incidences were analyzed in four studies [25, 26, 29, 33]. The studies that were assigned to the category Safety and Risk Group Analysis were mostly cohort studies, in addition to two case-control studies and one cross-sectional cohort study [35, 36]. Similar to the category Burden of Disease, predominantly time-to-event outcomes were evaluated using Kaplan-Meier estimation or Cox (proportional hazards) regression models [37, 40, 41, 43-45, 47-49]. In contrast, many studies have used propensity score methods such as matching and weighting [35, 37, 40, 43- 45, 48, 49] or applied the concept of target trial emulation [35, 40, 48]. Similar to the category Safety and Risk Group Analysis, most studies in the Treatment Comparison scenario examined time-to-event outcomes. They employed propensity score methods such as matching and weighting or applied the concept of target trial emulation. In addition to that, two studies used the clone method, a special form of emulating a target trial where each individual is cloned into both treatment groups, censored patients according to their designated treatment strategy and weighted the uncensored to avoid selection bias [13, 54, 59].

Table S4 Overview of study characteristics and limitations

| Author | Study Design | Aim of Study | Outcome | Methods | Scope | Countries | Limitations | | | | | | | | |
| --- | --- | --- | --- | --- | --- | --- | --- | --- | --- | --- | --- | --- | --- | --- | --- |
|  |  |  |  |  |  |  | Main Bias Categories | | | | Additional Bias Category | | | | |
| Burden of disease | | | | | | | Confounding | Selection bias | Information bias | Reporting bias | Follow-up Challenges | Missing Data | Coding Challenges | Validation & Data quality | Operationalization or availability of variables |
| Beck et al. [26] | cohort study | genetic association | prevalence | Poisson test | multi-center | USA |  | x | x |  |  |  | x |  |  |
| Biccler et al. [31] | cohort study | relapse risk and loss of lifetime after treatment | relapse risks, survival | logistic regression, KM estimation, Aalen-Johansen estimation | multi-national | Nordic countries | x |  |  |  | x |  |  | x |  |
| Canavan et al. [23] | cohort study | association of EOL treatment and practice-level factors | use of EOL therapy | adjusted logistic regression with random intercept | nation-wide | USA | x | x | x | x |  | x |  |  | x |
| Chang et al. [33] | cohort study | genetic association | prevalence | adjusted Firth logistic regression | multi-center | USA |  | x | x | x |  |  |  | x |  |
| Coltin et al. [32] | cohort study | burden of surviving disease | cumulative incidence of mortality | time-to-event analyses, Cox model | multi-center | Canada |  | x |  |  | x |  |  |  |  |
| Forrest et al. [27] | cohort study | disease-predictive ML | disease prediction | random forest model, model evaluation: AUROC, Sensitivity, Specificity, PPV, NPV, Brier score, adj. linear -, logistic -, and Cox regression | nation-wide | UK |  | x | x |  |  | x |  |  |  |
| Haas et al. [29] | cohort study | impact and effectiveness of treatment | prevalence, incidence rate ratio | negative binomial regression | nation-wide | Israel | x | x | x | x |  |  |  |  |  |
| Kamran et al. [24] | cohort study | ML model development | mortality, treatment events | regularized logistic regression, AUROC, model calibration | multi-center | USA | x |  |  |  |  |  |  |  | x |
| Manz et al. [30] | cohort study | Validation of ML Algorithm | mortality | AUROC, AUPRC, scaled Brier score, logistic regression | multi-center | USA |  |  | x |  |  | x | x |  |  |
| Vasileiou et al. [28] | cohort study | hospital admission after vaccination | hospital admission | time-dependent Cox model & Poisson regression with inverse propensity weights | nation-wide | UK | x | x |  |  | x |  |  |  |  |
| Witberg et al. [25] | cohort study | frequency and severity of disease after treatment | (cumulative) incidence | KM estimation | nation-wide | Israel |  | x | x |  |  | x |  |  |  |
| Safety and Risk Group | | | | | | | Confounding | Selection bias | Information bias | Reporting bias | Follow-up Challenges | Missing Data | Coding Challenges | Validation & Data quality | Operationalization or availability of variables |
| Berry et al. [47] | cohort study | genetic association | incidence, prevalence | Cox regression, adj. logistic regression | multi-center | USA UK | x | x | x |  |  |  |  | x |  |
| Chavez-MacGregor et al. [46] | cohort study | outcome comparison of risk groups | morality, ventilation, ICU stay, hospitalization | adj. logistic regression | multi-center | USA | x | x |  |  |  |  |  |  | x |
| Chemaitelly et al. [49] | cohort studies | treatment effectiveness among children and adolescents | incidence rate, cumulative incidence | 1:1 matching, KM estimation, Cox regression | nation-wide | Qatar | x | x |  | x |  |  |  |  |  |
| Cohen-Stavi et al. [35] | cohort study, case-control study | treatment effectiveness among children | cumulative incidence | target trail emulation, matching, KM estimation | multi-center | Israel | x |  | x |  | x |  |  |  |  |
| Damrauer et al. [36] | case-control study, cross sectional study | genetic association | prevalence | adj. logistic regressions | multi-center | USA |  | x | x |  |  | x | x |  |  |
| Deng et al. [48]* | cohort study | comparative effectiveness | time to event | target trail emulation, inverse probability of treatment weighting, generalized boosted models for PS, inverse probability of treatment weighted Kaplan-Meier method, PS-weighted Cox regression | nation-wide | USA | x | x | x |  | x | x |  |  | x |
| Filion et al. [37]* | cohort study | adverse event association | major adverse events | time conditional PS using conditional logistic regression, 1:1 matching, Cox regression | multi-center | Canada UK | x | x | x | x | x |  |  |  | x |
| Forrest et al. [38] | cohort study | disease risk associated with gene variants | risk difference between prevalence | 2-sided Fisher’s exact tests | multi-center | USA UK |  | x | x |  |  |  | x |  |  |
| Harstad et al. [51]* | observational study | treatment effectiveness | frequency, relative risk of Adverse Effects | logistic and Cox regression | multi-center | USA | x | x |  |  | x |  |  | x |  |
| Li et al. [39] | cohort study | adverse event association | incidence rate | descriptive analyses | multi-national | Australia France Germany Japan Netherlands Spain UK US |  |  | x | x |  | x |  | x |  |
| Lyu et al. [40]* | cohort study | comparative effectiveness | incidence | target trail emulation, PS- matching, Cox regression | nation-wide | UK | x |  | x |  | x |  |  |  |  |
| Martin et al. [41] | cohort study | therapy evaluation | time-to-next-treatment, overall survival | KM, Cox regression | nation-wide | USA | x |  | x |  |  | x |  |  | x |
| Seymour et al. [42] | cohort study | phenotype derivation | phenotype frequency, mortality | Multiple imputation, k- means clustering | single-center | USA |  | x | x |  |  | x |  |  |  |
| Song et al. [50] | cohort study | comparative effectiveness | disease risk | adj. logistic regression | nation-wide | USA | x |  |  | x |  |  |  |  |  |
| Suchard et al. [44]* | cohort study | comparative effectiveness | relative risk | PS model, matching, Cox regression, meta analysis | multi-national | USA South Korea Japan Germany | x | x | x | x |  |  |  |  |  |
| Vinogradova et al. [45] * | cohort study | treatment association | hospital admission or death | PS, Cox regression | nation-wide | UK | x |  | x | x |  |  |  |  | x |
| You et al. [43]* | cohort study | treatment association | net adverse clinical events | PS-matching, Cox regression | multi-center | South Korea USA | x |  | x | x |  |  |  | x | x |
| Treatment Comparison | | | | | | | Confounding | Selection bias | Information bias | Reporting bias | Follow-up Challenges | Missing Data | Coding Challenges | Validation & Data quality | Operationalization or availability of variables |
| Andersson et al. [57] | cohort study | comparative effectiveness | hospital admission, death | target trail emulation, PS-matching, inverse probability of treatment weights, KM | multi-national | Nordic countries | x | x | x |  |  |  |  |  |  |
| Deputy et al. [58] | case-control study | vaccine effectiveness | association between vaccination status and case patient or control patient status | matching, conditional logistic regression | nation-wide | USA | x | x | x |  |  |  |  |  |  |
| Kim et al. [53] | cohort study | treatment effectiveness | time to cardiovascular events | PS-matching, stratified Cox regression | nation-wide | South Korea | x | x | x | x |  |  |  |  |  |
| Mahévas et al. [55] | case-control study | treatment effectiveness | survival | inverse probability of treatment weighting, Cox regression, KM, multiple imputation | multi-center | France | x | x |  |  |  |  |  |  | x |
| Marafino et al. [62] | cohort study | intervention association | mortality, readmission | target-trail emulation, generalized linear mixed effects models, difference-in-difference analysis | multi-center | USA | x | x |  |  |  |  | x |  |  |
| Rentsch et al. [56] | cohort study | early treatment association | mortality | inverse probability of treatment weighted KM, Cox regression | nation-wide | USA | x | x | x |  |  | x |  | x | x |
| Wang et al. [12] | cohort study | RCT comparison | time to event | target trail emulation, PS matching, Cox regression | nation-wide | USA | x | x |  | x | x |  |  |  |  |
| Wong et al. [61] | cohort study, case-control study | comparative effectiveness | mortality, hospital admission, progression | propensity score matching, Cox regression, conditional logistic regression | territory-wide | Hong Kong | x | x | x |  |  | x |  |  |  |
| Xie et al. [54] | cohort study | treatment association | relative risk, event rate, absolute risk reduction:  hospital admission, death | target trail emulation, clone method, inverse probability of censoring weight | nation-wide | USA | x |  | x |  |  |  |  |  |  |
| Xie et al. [59] | cohort study | treatment effectiveness | relative risk, event rate, absolute risk reduction: hospital admission, death | target trail emulation, inverse probability weighting, clone method, weighted KM | nation-wide | USA | x | x | x |  |  |  |  |  |  |
| Zheng et al. [60] | cohort study | comparative effectiveness | hospital admission, death | Cox regression, PS- weighting | nation-wide | UK | x | x | x |  |  |  |  |  | x |

Abbreviation: KM… Kaplan-Meier, PS... Propensity Score, EOL… End-of-Life, ICU… Intensive Care Unit, ML… Machine Learning, AUROC… Area under the receiver operating curve, AUPRC… Area under the precision-recall curve

* This indicates publications that were not uniquely categorizable into the main scenarios. Specifically, they did not only compare the effectiveness of treatments but compared the risk of adverse events between two or more treatments
